# Neural state reduction: a quantum-inspired framework for how latent conscious thought becomes reportable

**DOI:** 10.3389/fncom.2026.1865388

**Published:** 2026-09-17

**Authors:** A. K. Pradeep

**Affiliations:** Sensori.Ai, Piedmont, CA, United States

**Keywords:** cognitive modeling, computational neuroscience, consciousness, Hilbert space, introspection, metacognition, neural dynamics, quantum cognition

## Abstract

Human conscious thought often appears as a diffuse, unstable, and only partly verbalizable field until attention, questioning, decision, or speech stabilizes it into a reportable form. This manuscript develops a quantum-inspired neural framework for that transition. The proposal does not claim that consciousness literally depends on microscopic quantum computation, that the brain implements a physical quantum computer, or that neuronal tissue maintains macroscopic quantum coherence. Instead, it uses the mathematical architecture of quantum theory—state vectors, density matrices, projection operators, non-commuting measurements, interference, decoherence-like contextual stabilization, and state reduction—as a formal language for modeling the transformation from latent conscious potential to reportable thought. In the proposed framework, pre-report consciousness is represented as a high-dimensional latent neural-cognitive state over candidate thoughts. This state evolves under a neural-cognitive Hamiltonian shaped by memory, salience, affect, attention, language, task demands, and spontaneous associative dynamics. A question such as “What are you thinking about?” is modeled as a measurement context that selects a basis and projects the latent state into a reportable cognitive eigenstate. The framework, therefore, treats an introspective report not as a passive readout of a pre-existing mental object but as an active state-reducing operation. Illustrative simulations demonstrate three formal consequences: (1) Non-commuting introspective questions generate order effects; (2) selective reporting reduces candidate-state entropy and suppresses off-diagonal latent coherence; and (3) overlapping candidate thoughts can produce interference-like probability structures. The model generates testable predictions for experience sampling, electroencephalography (EEG)/magnetoencephalography (MEG)/functional magnetic resonance imaging (fMRI) representational decoding, intracranial recordings, and perturbation studies: Introspective reports should depend on question order; spoken reports should reduce latent-state entropy more strongly than silent attention; ambiguous thought states should exhibit interference-like probability signatures; and neural activity should transition from high-dimensional latent dynamics toward lower-dimensional, report-stabilized states. Neural state reduction provides a computational bridge among quantum cognition, consciousness science, metacognition, global access, and the neuroscience of reportability.

## 1 Introduction

A familiar feature of conscious life is that thought is often not already present as a single sentence waiting to be spoken. Before reporting, conscious mentation may feel like a field of partially formed possibilities, including images, feelings, semantic fragments, bodily states, memories, intentions, and anticipations. When another person asks, “What are you thinking about?” or when one asks this question of oneself, that field is often compressed into one answer. The reported thought may be honest and useful, but it is not necessarily a transparent extraction of a determinate pre-existing object. Rather, the act of asking may alter the very state being queried.

This study proposes a formal framework for modeling that transition. The guiding hypothesis is that conscious thought before report is usefully modeled as a high-dimensional latent neural-cognitive state containing multiple partially activated candidate thoughts. A report demand—whether induced by attention, introspection, questioning, decision, or speech—imposes a context-specific measurement basis and reduces that latent state to a reportable cognitive state. The framework is called neural state reduction.

The analogy is motivated by quantum theory but does not require the claim that consciousness is physically quantum. In the double-slit experiment, the interference pattern exhibits a wave-like probability structure, while measurement of which path the particle took destroys the interference and yields a particle-like outcome. In standard quantum mechanics, the Schrödinger equation describes continuous unitary evolution, whereas collapse or state reduction enters through the measurement postulate, the Born rule, decoherence theory, or interpretation-specific additions. This distinction is crucial. The present theory does not claim that the Schrödinger equation itself explains introspective collapse, nor that neuronal ensembles implement literal quantum collapse. Rather, it borrows the mathematical distinction between continuous latent-state evolution and measurement-context-dependent state reduction.

The proposal sits within an established scientific lineage. Quantum cognition has shown that the mathematics of Hilbert spaces, incompatible observables, contextual probability, interference, open-system dynamics, and sequential measurement can model cognitive phenomena that are awkward for classical probability, including question-order effects, conjunction and disjunction effects, ambiguous perception, similarity, conceptual combination, and decision under uncertainty (Busemeyer and Bruza, 2024; Busemeyer and Lu, 2025; Khrennikov, 2010; Pothos and Busemeyer, 2013; Yearsley and Busemeyer, 2016). This literature generally does not claim that the brain is a microscopic quantum computer. The main claim is formal: Quantum probability provides a mathematically coherent alternative to classical probability when cognitive measurements are contextual, order-dependent, and state-changing.

Consciousness science supplies the second foundation. Global neuronal workspace theory emphasizes that conscious access involves ignition, amplification, and availability of information to distributed systems, including systems supporting report, action, reasoning, and memory (Baars, 1988; Dehaene and Changeux, 2011; Mashour et al., 2020). Integrated information theory emphasizes the structure and integration of conscious experience (Tononi et al., 2016). No-report paradigms have shown that report can confound the neural correlates of consciousness because the neural machinery of reporting may be partly distinct from the neural machinery of experiencing (Tsuchiya et al., 2015). Studies of ongoing thought show that spontaneous experience is heterogeneous, context-dependent, and distributed across multiple neural systems, including default-mode, attention, and control networks (Smallwood et al., 2021). Metacognition research further suggests that reports about one’s own mental states are cognitive achievements rather than direct, effortless readouts (Fleming and Dolan, 2012; Overgaard and Sandberg, 2012).

Recent working-memory theories provide a complementary cognitive architecture for the latent-to-reportable transition. In the embedded-process view, a broad activated portion of long-term memory contains currently accessible representations, while a capacity-limited focus of attention holds a small subset under joint bottom-up and top-down control (Cowan et al., 2025). A convergent multicomponent account places a consciously accessible, multidimensional “awareness buffer” at the focal point of working memory, integrating storage, executive manipulation, and attentional control (Allen et al., 2026). Neural state reduction does not replace either account. Instead, it supplies a formal model for how a question or report demand can change the relevant selection basis and stabilize one report from a wider set of activated, attended, and contextually available representations.

The central claim of this study is therefore specific and limited: Conscious report can be mathematically modeled as a measurement-like state reduction imposed on a high-dimensional latent neural-cognitive state. This claim has three advantages. First, it explains why introspective questioning may change the mental state being reported. Second, it offers a formal vocabulary for ambiguity, interference, and order effects in conscious thought. Third, it generates empirically testable predictions using current computational neuroscience tools. The novelty lies not in asserting that cognition is physically quantum but in treating the act of reporting itself as a context-dependent state-changing operation.

## 2 Conceptual boundary: quantum-inspired, not quantum brain

The most important boundary condition is: negative: This manuscript does not argue that the brain uses microscopic quantum computation to generate consciousness. It does not require long-lived quantum coherence in microtubules, synapses, ion channels, or cortical tissue. It does not claim that neural firing violates classical biophysics. The argument is formal and computational.

The term *quantum-inspired* is used here in the same sense as it is commonly used in quantum cognition: The mathematical structures of quantum theory are used to model contextual, order-dependent, and state-changing cognitive processes (Busemeyer and Bruza, 2024; Busemeyer and Lu, 2025). The state vector is not a physical wave function in space. The Hilbert space is not the physical configuration space of a particle. The measurement operator is not a photodetector. The Born-like probability rule is not a claim that Planck’s constant governs thought. Instead:

- A state vector represents latent cognitive potential;
- A density matrix represents a noisy, mixed, context-sensitive neural-cognitive state;
- A basis represents the set of distinctions made available by a question or task;
- A projection operator represents attentional, linguistic, or decisional selection;
- Non-commutativity represents the fact that one question can change the state to which the next question is applied;
- Interference represents the non-classical combination of overlapping meanings or candidate thoughts;
- Decoherence-like stabilization represents the suppression of latent ambiguity by attention, language, report, and action.

This boundary is essential for conceptual clarity. A paper claiming literal quantum consciousness would face a much higher evidentiary burden. A paper claiming that quantum probability supplies a useful formal language for reportable thought is more defensible within existing quantum cognition and computational neuroscience.

## 3 Background

### 3.1 From quantum measurement to cognitive measurement

In quantum theory, a system can be represented by a state vector

$$|\Psi_{c}(t)\langle =\sum_{i}c_{i}(t)|\tau_{i}\rangle ,$$ (1)

Where the coefficients encode probability amplitudes. Prior to measurement, the state can contain coherent superpositions over possible outcomes. Under unitary evolution, the state evolves continuously according to a Schrödinger-type equation. Measurement is different: It imposes an observable, selects a basis, and yields an outcome with probability given by the Born rule. After a selective measurement, the state is updated according to the observed outcome.

In cognition, the analogy is not that thought is a photon. The analogy is that both physical measurement and cognitive questioning involve a transition from latent potential to context-defined outcome. The question “What are you thinking?” does not merely read a scalar variable. It creates a report space. It asks the person to select a basis: thought, feeling, image, memory, plan, worry, desire, or word. Different questions impose different bases. These bases may be incompatible in the technical sense that applying one first changes the probabilities associated with another.

Quantum cognition has used this principle to model question-order effects in surveys and judgments. If two questions correspond to non-commuting observables, the order of questioning can change the distribution of answers. This study extends that principle to conscious thought report: Introspective questions may not only sample mental content but also shape the state from which subsequent reports are drawn.

### 3.2 Conscious access, report, and the problem of measurement

Consciousness research has long depended on reports. A subject presses a button, speaks a percept, rates confidence, or describes an experience. Yet the report is not neutral. It requires attention, working memory, metacognitive access, response selection, motor planning, and often language. No-report paradigms attempt to separate the neural basis of experience from the neural basis of reporting, precisely because report-related activity can contaminate the search for the neural correlates of consciousness (Tsuchiya et al., 2015). This creates a conceptual tension: a report is necessary for much of consciousness science, but the act of reporting may also change the system being measured.

Neural state reduction treats this tension as central rather than incidental. The report is not a nuisance variable added after experience. It is a transformation. An unreported cognitive state may contain a distribution of latent contents and partially stabilized meanings. A report demand imposes a representational constraint, suppresses alternatives, and produces a stabilized outcome. This does not mean that the reported thought is false. It means that the reported thought is the result of a cognitive measurement process.

### 3.3 Activated memory, focus of attention, and reportable selection

The embedded-process view provides a concrete cognitive architecture for the candidate states in this framework. Cowan et al. (2025) distinguish the temporarily activated portion of long-term memory from a capacity-limited focus of attention embedded within it. Activated memory includes retrieved knowledge, newly learned episodes, concepts, and bindings that are accessible without all being simultaneously focal. A small subset enters the focus of attention, guided partly by executive control and partly by bottom-up salience, novelty, or thematic relevance. The contents of the focus can then be integrated, deliberately manipulated, and used for a report.

This architecture maps naturally, but not identically, onto neural state reduction. The broader latent state includes activated representations that are available to influence a report, while a question or task helps define which distinctions become relevant and which subset is stabilized. The projection operation is therefore not simply a synonym for attention. Rather, it formalizes the context-sensitive selection and updating that occurs when activated and attended contents must be converted into an explicit answer. Some candidate components may already be consciously experienced but diffuse or pre-linguistic; others may be activated-memory states outside the current focus that bias what becomes reportable. The framework is therefore agnostic about whether every latent candidate is phenomenally conscious before reporting.

The convergence is strengthened by Allen et al. (2026), who reformulated the episodic buffer as an “awareness buffer”: a consciously accessible, multidimensional interface that combines storage with executive manipulation and attentional control. The embedded focus of attention and the awareness buffer describe cognitive bottlenecks through which distributed information becomes available for flexible behavior. Neural state reduction adds a complementary mathematical question: How does the basis imposed by a particular probe, decision, or linguistic demand alter the probabilities and subsequent state of the contents that pass through that bottleneck?

This field-to-focus structure also has a historical lineage. Cowan and Rachev (2018) showed that Wilhelm Wundt’s account of consciousness anticipated important features of modern embedded-process theories, including a broad field of activated content and a narrower focus associated with apperception and introspection. Neural state reduction does not claim to have originated the field-to-focus distinction. Its contribution is to express the transition in an explicit state-space language that supports contextuality, non-commutativity, interference, and falsifiable comparisons between models.

### 3.4 Questioning, reconstructive memory, and report formation

The effects of questioning are not confined to immediate introspection. Research on reconstructive memory shows that the wording of a probe can alter later reports about an event. In the classic experiments of Loftus and Palmer (1974), verbs used in questions about automobile collisions changed speed estimates and influenced later reports of broken glass that had not been present. This study provides an important behavioral boundary condition for neural state reduction: A measurement context can stabilize an inaccurate or reconstructed report, rather than only select among equally veridical alternatives.

This point sharpens the theory. State reduction is not an epistemic guarantee. A report-stabilized state is the output of interactions among latent representations, memory, attention, language, expectations, and task demands. Leading wording, social demand, and source-monitoring failure can therefore alter the state that becomes reportable. Such effects are compatible with classical reconstructive-memory models and do not by themselves establish quantum-like cognition. The distinctive empirical burden for neural state reduction is to show that basis dependence, order effects, or non-additive probability structure improves prediction beyond those classical accounts.

### 3.5 Ongoing thought as high-dimensional latent dynamics

Ongoing conscious thought is not a single process. It includes spontaneous memory, future simulation, task-related planning, imagery, emotion, rumination, embodied sensation, and linguistic rehearsal. Contemporary work on ongoing thought suggests that its content and dynamics are shaped by the interaction of internal state, task context, attention, control, and default-mode processes (Smallwood et al., 2021). This is exactly the kind of system for which a high-dimensional state-space description is appropriate.

A classical approach might represent thought as a probability distribution over discrete categories that is useful but incomplete when mental states are ambiguous, partially overlapping, and changed by the act of inquiry. A Hilbert-space representation adds three features: (1) basis dependence, indicating that the same latent state can be described differently under different questions; (2) interference, indicating that candidate thoughts can combine non-additively; and (3) non-commutativity, indicating that the order of cognitive operations matters.

## 4 Formal theory

### 4.1 Cognitive state space

Let ${ℋ}_{C}$ denotes a Hilbert-like cognitive state space. The basis vectors $|\tau_{i}\rangle$ represent candidate reportable thought states. These states may correspond to semantic propositions, images, feelings, memories, plans, action tendencies, or mixtures thereof. The cognitive state-space representation is given in Equations 1–3:

$$|\Psi_{C}(t)\langle =\sum_{i=1}^{N}c_{i}(t)|\tau_{i}\rangle$$ (2)

Where $c_{i}(t)$ are cognitive amplitudes, and the squared magnitude of $c_{i}(t)$ represents the probability that candidate thought i becomes reportable under a compatible measurement context. The state is normalized:

$$\Psi_{C}(t)|\Psi_{C}(t)=1$$ (3)

This formulation does not imply that the brain literally stores complex numbers. It means that the formal model requires amplitudes and phases to represent contextual interference among candidate thoughts.

### 4.2 Neural-cognitive Hamiltonian

The continuous pre-report evolution is described by Equation 4:

$$i\tau_{C}\frac{\partial }{\partial t}|\Psi_{C}(t)\langle ={\hat{H}}_{C}(t)|\Psi_{C}(t)\rangle$$ (4)

Here $\tau_{C}$ is a cognitive timescale constant rather than Planck’s constant. ${\hat{H}}_{C}(t)$ is a neural-cognitive Hamiltonian that governs the flow of activation through the latent thought space. It can be decomposed as:

$$\begin{array}{l} {\hat{H}}_{C}(t)={\hat{H}}_{0}+{\hat{H}}_{\text{memory}}+{\hat{H}}_{\text{salience}}\\ +{\hat{H}}_{\text{affect}}+{\hat{H}}_{\text{attention}}+{\hat{H}}_{\text{language}}+{\hat{H}}_{\text{task}} \end{array}$$ (5)

The terms have the following interpretation:

-
${\hat{H}}_{0}$ : spontaneous associative dynamics;
-
${\hat{H}}_{\text{memory}}$ : episodic, semantic, autobiographical, and procedural constraints;
-
${\hat{H}}_{\text{salience}}$ : motivational and relevance weighting;
-
${\hat{H}}_{\text{affect}}$ : emotional, interoceptive, and bodily modulation;
-
${\hat{H}}_{\text{attention}}$ : gain, focus, and selection dynamics;
-
${\hat{H}}_{\text{language}}$ : linguistic compressibility and reportability;
-
${\hat{H}}_{\text{task}}$ : current external or internal goal constraints.

Equation 5 is not a literal physical law of cortical tissue. It is a compact computational statement: the latent neural-cognitive state evolves under multiple interacting constraints until a measurement-like event stabilizes one reportable outcome.

### 4.3 Density matrix formulation

The density-matrix representation and its open-system evolution are given in Equations 6, 7:

$$\rho_{C}(t)=\sum_{j}p_{j}|\Psi_{j}(t)\rangle \;\langle \Psi_{j}(t)|$$ (6)

The diagonal terms $\rho_{\mathrm{ii}}$ represent activation probabilities of candidate thought states in a chosen basis. The off-diagonal terms $\rho_{\mathrm{ij}}$ , for i≠j , represent contextual coherence among candidate thoughts, including semantic blending, unresolved ambiguity, affective mixtures, metaphorical overlap, and partially formed associations.

The open-system evolution can be written as:

$$\frac{d\rho_{C}}{\mathrm{dt}}=-\frac{i}{\tau_{C}}[{\hat{H}}_{C},\rho_{C}]+\sum_{k}\gamma_{k}({\hat{L}}_{k}\rho_{C}{\hat{L}}_{k}^{†}-\frac{1}{2}\{{\hat{L}}_{k}^{†}{\hat{L}}_{k},\rho_{C}\})$$ (7)

The first term represents coherent latent associative evolution. The second term is a Lindblad-style contextual stabilization term. The operators ${\hat{L}}_{k}$ represent environmental, attentional, linguistic, bodily, or social constraints that reduce ambiguity and stabilize one or more candidate states. The rates $\gamma_{k}$ represent the strength of these stabilizing influences.

### 4.4 Question as measurement context

A question Q defines a measurement context. It does so by specifying which distinctions matter. “What are you thinking?” differs from “What are you feeling?”; “What do you want?” differs from “What do you fear?”; “What should we do?” differs from “What do you remember?” Each question defines a basis or set of subspaces in ${ℋ}_{C}$ .

Let the question Q be represented by a set of projection operators. The measurement context and corresponding report probability are defined in Equations 8, 9:

$$\{{\hat{\Pi }}_{Q,1},{\hat{\Pi }}_{Q,2},\ldots ,{\hat{\Pi }}_{Q,K}\},\;\;\;\;\sum_{r=1}^{K}{\hat{\Pi }}_{Q,r}=\hat{I}$$ (8)

The probability of report r under question Q is:

$$P(R=r|Q)=\mathrm{Tr}({\hat{\Pi }}_{Q,r}\rho_{C}{\hat{\Pi }}_{Q,r})$$ (9)

After a selective report, the state is updated by a Lüders-type rule:

$$\rho_{C}\to \rho_{C|Q,r}=\frac{{\hat{\Pi }}_{Q,r}\rho_{C}{\hat{\Pi }}_{Q,r}}{\mathrm{Tr}({\hat{\Pi }}_{Q,r}\rho_{C})}$$ (10)

Equation 10 is the formal heart of neural state reduction. The reported thought is not merely retrieved unchanged; it is selected and stabilized by the interaction between the latent state and the measurement context.

### 4.5 Non-commuting introspective questions

Two questions $Q_{A}$ and $Q_{B}$ are non-commuting if their measurement operators do not commute:

$$[{\hat{M}}_{Q_{A}},{\hat{M}}_{Q_{B}}]={\hat{M}}_{Q_{A}}{\hat{M}}_{Q_{B}}-{\hat{M}}_{Q_{B}}{\hat{M}}_{Q_{A}}\neq 0$$ (11)

The non-commutativity defined in Equation 11 implies the order effect expressed in Equation 12:

$$P(R_{B}|Q_{A}\to Q_{B})\neq P(R_{B}|Q_{B}\to Q_{A})$$ (12)

For conscious reporting, this predicts that asking “What are you feeling?” before asking “What are you thinking?” can produce a different thought report than asking those questions in the reverse order. The first question changes the state to which the second question is applied.

### 4.6 Interference among candidate thoughts

If two candidate thoughts A and B overlap semantically or affectively, their combined probability does not need to be classically additive. Interference among candidate thoughts is represented by Equation 13:

$$P(A\cup B)=P(A)+P(B)+2\sqrt{P(A)P(B)}\cos\theta$$ (13)

Where θ is a relative semantic phase. Constructive interference occurs when candidate thoughts reinforce each other; destructive interference occurs when they inhibit or cancel each other. This provides a formal language for ambiguous states such as “I am both excited and anxious,” “I am thinking about the meeting, but really about my status,” or “I do not know what I think until I say it.”

## 5 Neural interpretation

The model can be mapped onto current neuroscience without requiring literal quantum biophysics. The latent state $\rho_{C}$ corresponds to distributed neural population activity and other temporarily accessible neural-cognitive representations across cortical and subcortical systems. Candidate thought states $|\tau_{i}\rangle$ correspond to metastable neural manifolds, attractor states, semantic embeddings, activated memory representations, or task-relevant representational subspaces. The Hamiltonian terms correspond to recurrent cortical dynamics, memory retrieval, affective and interoceptive salience, attentional gain, executive control, and linguistic constraints. Projection operators correspond to context-conditioned attentional selection, working-memory stabilization, verbal encoding, decision selection, and motor/report preparation.

The embedded-process mapping makes this interpretation more precise. The activated portion of long-term memory can supply a broad set of candidate representations; the capacity-limited focus of attention can hold a small subset; and bottom-up thematic draw, together with top-down executive control, can alter which subset becomes focal (Cowan et al., 2025). Neural state reduction models the additional transformation required when the system must answer a particular question, make a decision, or formulate a public report. In this sense, the cognitive bottleneck and the measurement basis are complementary constructs: The cognitive bottleneck limits what can be actively integrated, while the measurement basis defines the distinctions under which an explicit answer is selected.

In this mapping, conscious report is a form of neural stabilization. It may involve prefrontal control, parietal attention systems, language networks, default-mode and semantic systems, thalamo-cortical integration, and motor output systems. A reportable thought emerges when one region of the latent state space becomes sufficiently stabilized for access, verbalization, and action. This is compatible with global-access theories and awareness-buffer accounts but adds a specific mathematical description of the transition from context-sensitive multiplicity to reportable singularity.

The model does not say that all latent candidates are conscious, nor that only reported contents are conscious. The latent state is a computational state over report-relevant possibilities. Some components may be phenomenally experienced but diffuse, pre-linguistic, or only partly stabilized; others may be activated memory representations outside the current focus that influence selection without themselves being focal. Report-stabilized consciousness is the reduced state produced when the system must answer, decide, or speak.

## 6 Illustrative simulations

### 6.1 Purpose and status of the simulations

The simulations below are not presented as biological recordings or as fitted models of a specific dataset. They are conceptual and reproducible demonstrations of formal consequences implied by the model. Their purpose is to show that neural state reduction yields quantitative predictions that can later be tested with empirical neural data.

All simulations used synthetic, model-generated data. No human, animal, clinical, proprietary, or empirical neural datasets were used. The random seed was set to 42. The simulations were implemented in Python using NumPy and Matplotlib.

### 6.2 Simulation 1: non-commuting questions and introspective order effects

A two-dimensional cognitive state was initialized as a pure state biased toward one answer on a “thought” basis. A second “feeling” question was represented as a rotated basis. The simulation compared the probability of answering “thought = yes” directly with the probability of answering “thought = yes” after first applying the feeling-question measurement and ignoring its outcome. As the angular separation between the question bases increased, the order effect increased. The maximum absolute order-effect difference in this illustrative run was 0.4173 (Figures 1, 2).

**Figure 1:**
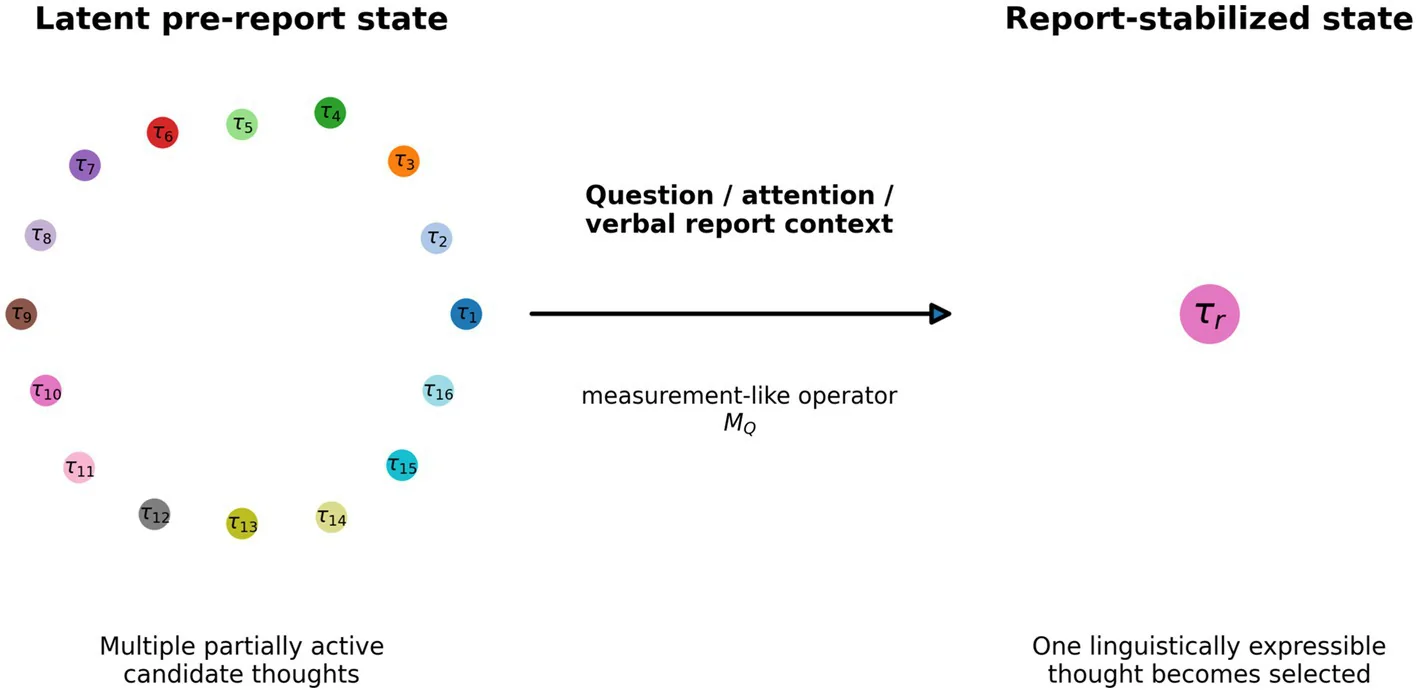
Conceptual schematic of neural state reduction. A diffuse latent pre-report state contains multiple partially active candidate thoughts. A question, attention, or verbal report context acts as a measurement-like operator that stabilizes one reportable state.

**Figure 2:**
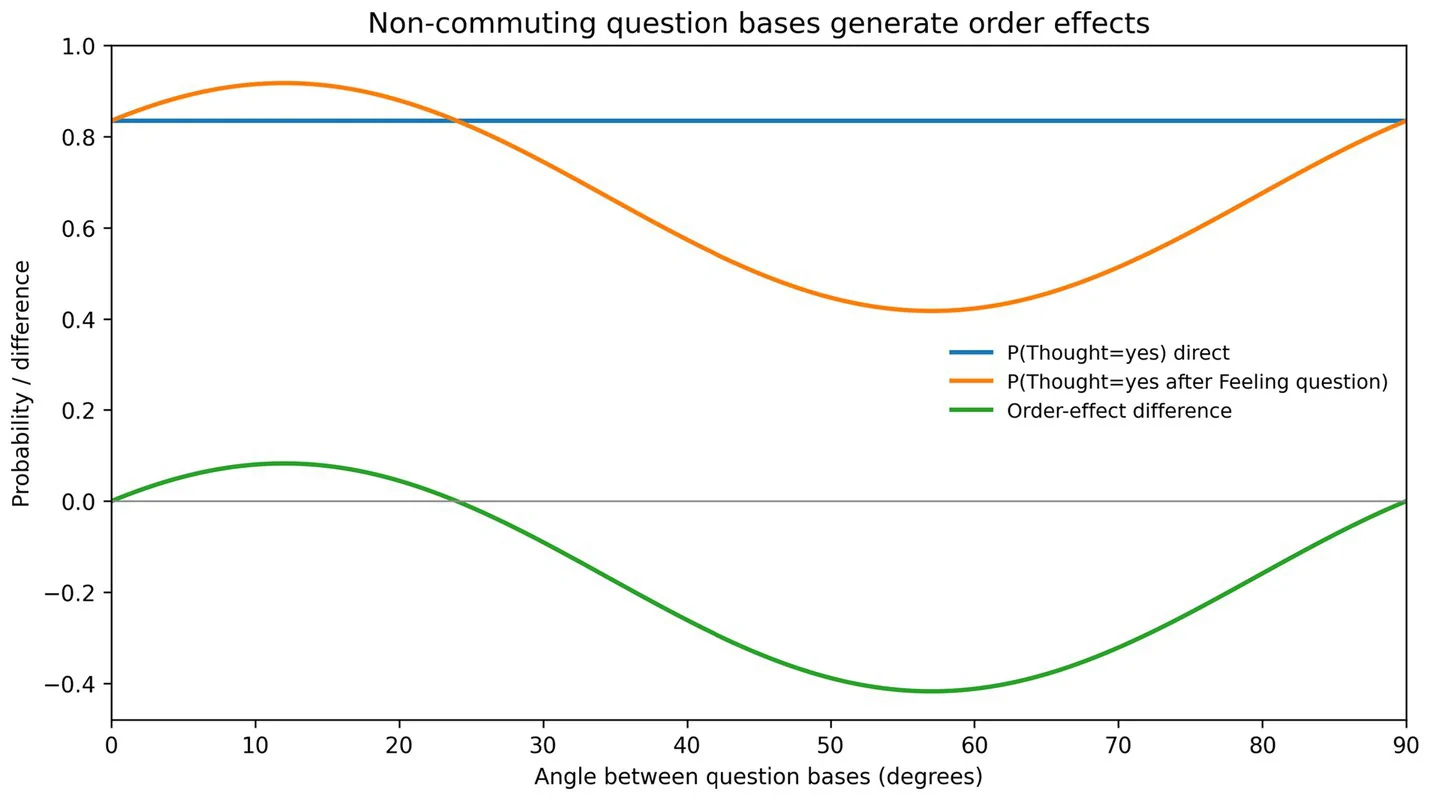
Non-commuting question bases generate order effects. The probability of endorsing a thought report changes when a feeling-question measurement is applied first, even when the outcome of the first question is ignored.

### 6.3 Simulation 2: report reduces candidate-state entropy

An eight-dimensional latent state was simulated across 5,000 Monte Carlo trials. Each trial contained a random complex candidate-amplitude vector mixed with isotropic noise to approximate an open neural-cognitive state. Candidate-state entropy was measured from the diagonal probabilities in the report basis. A selective report was then sampled according to the Born-like probability rule, and the state was updated to the selected report state. The pre-report candidate-state entropy was 2.6554 bits with a 95% confidence interval of 0.0043 bits. The post-report selected state had zero candidate-state entropy by construction. The mean off-diagonal coherence magnitude reduced from 0.0801 to zero.

This result is not intended to show that real brains become zero-entropy systems after the report. Rather, it illustrates the model’s operational claim: Selective reporting should reduce the dimensionality and ambiguity of the report-relevant state space. Empirical tests should expect partial rather than complete entropy reduction (Figures 3, 4).

**Figure 3:**
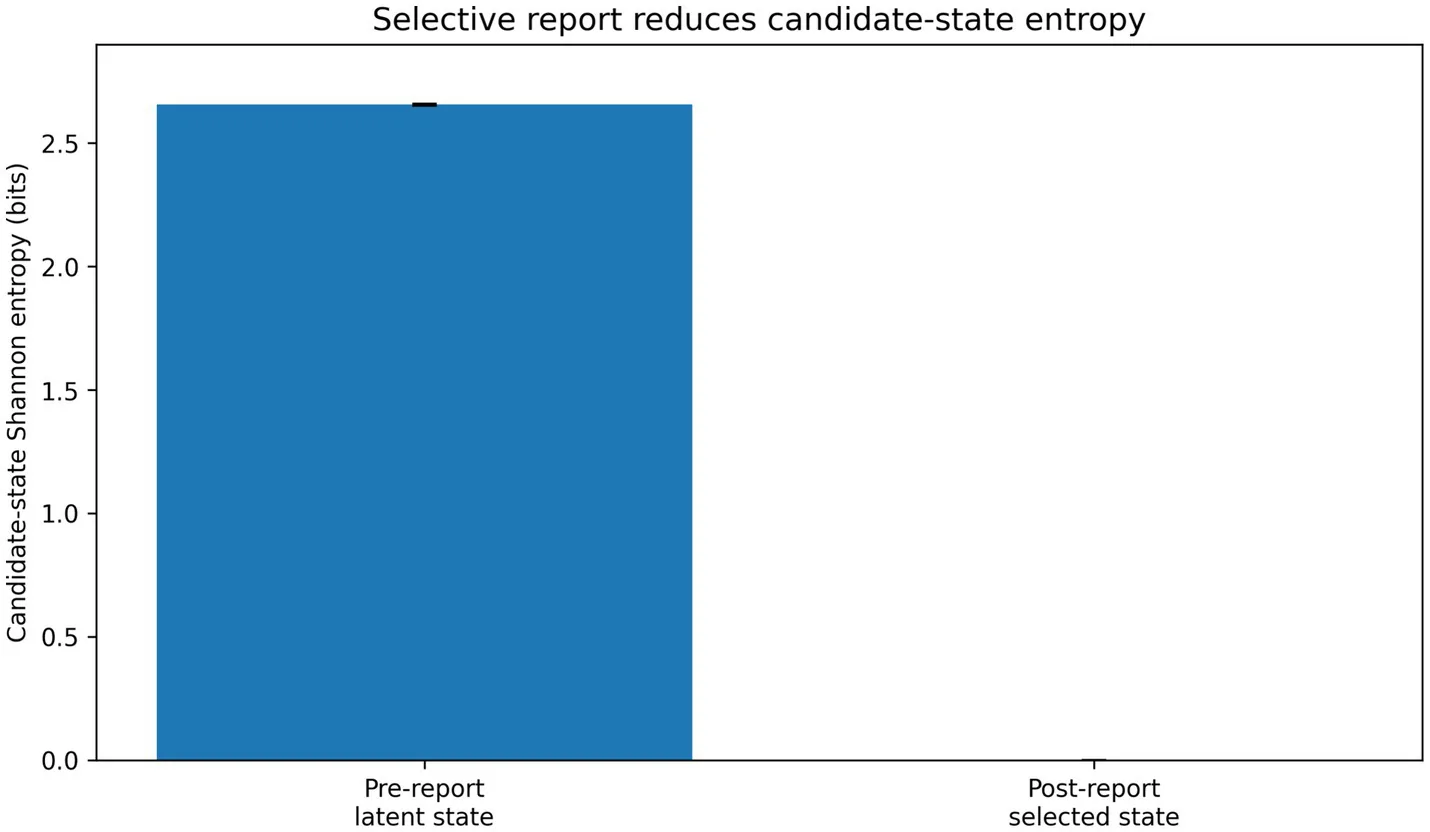
Selective report reduces candidate-state entropy in a synthetic eight-state latent thought model. Error bars represent 95% confidence intervals across 5,000 Monte Carlo trials.

**Figure 4:**
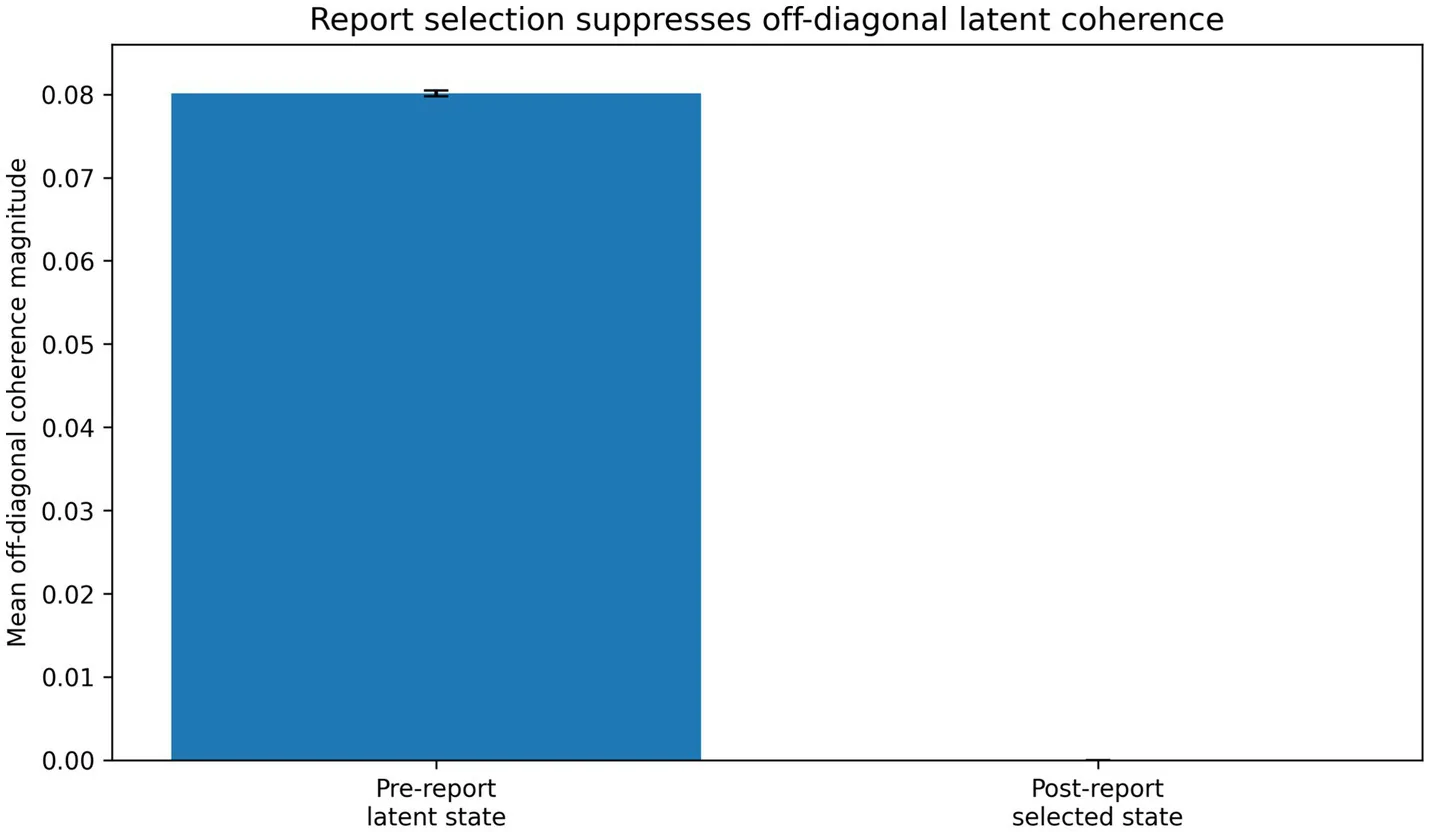
Report selection suppresses off-diagonal latent coherence in the report basis. In empirical neural data, the predicted effect is partial suppression rather than literal elimination.

### 6.4 Simulation 3: interference among ambiguous candidate thoughts

The third simulation compared a classical additive probability benchmark with a quantum-like contextual probability containing an interference term. Two candidate thoughts A and B were assigned baseline probabilities P(A)=0.34 and P(B)=0.24 . The relative semantic phase θ was varied from 0 to 2π . The resulting report probability ranged from near suppression to saturation, depending on the interference term. This demonstrates how overlapping candidate thoughts can combine non-additively (Figure 5).

**Figure 5:**
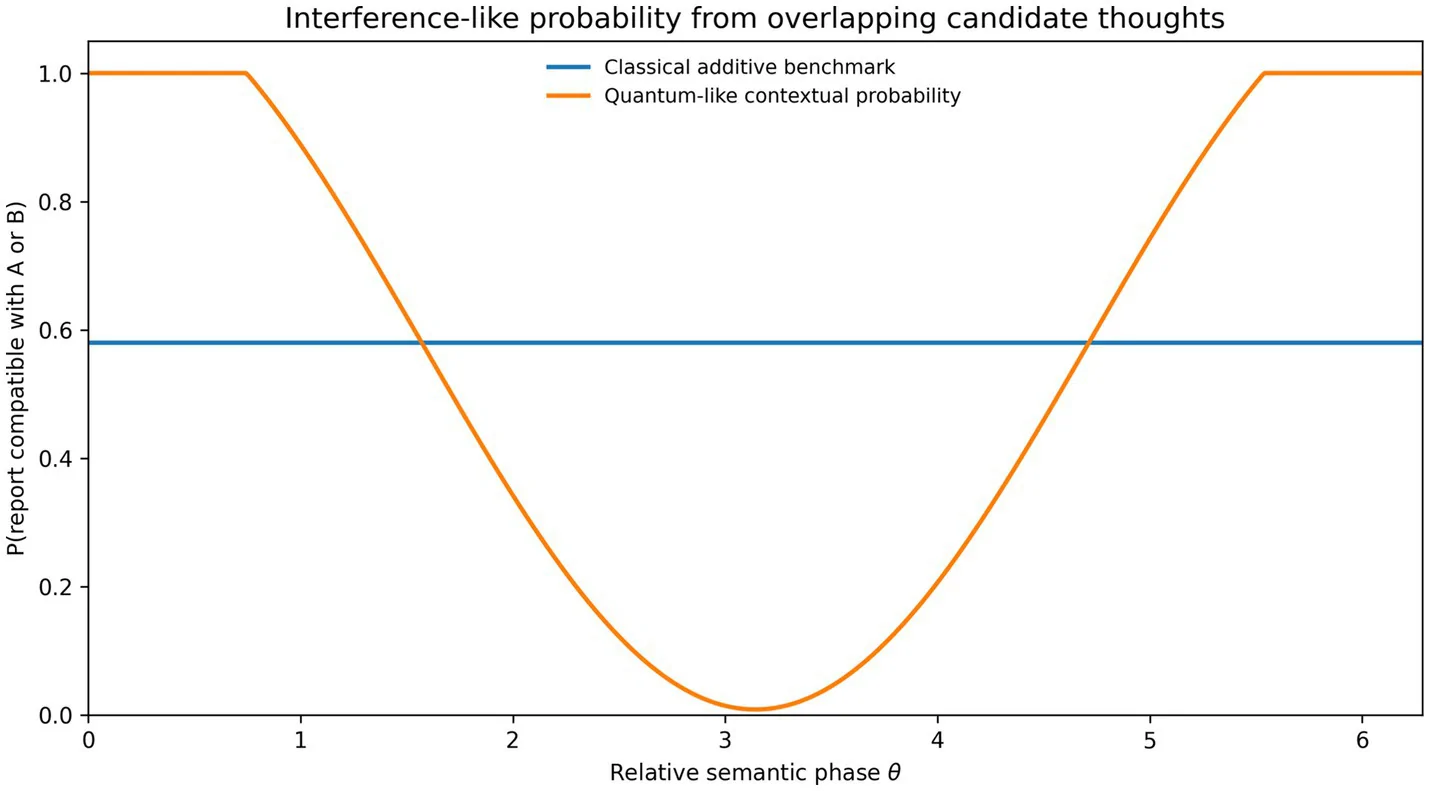
Interference-like probability from overlapping candidate thoughts. The quantum-like model departs from the classical additive benchmark when candidate thoughts are contextually related.

## 7 Predictions

### 7.1 Prediction 1: introspective question-order effects

If introspective questions impose non-commuting measurement contexts, reports should depend on question order. Asking “What are you feeling?” before “What are you thinking?” should not be equivalent to asking the same questions in reverse order. This can be tested in experience-sampling studies by randomizing introspective question order and comparing the distribution of reports.

### 7.2 Prediction 2: spoken report should reduce latent-state entropy more than silent attention

The model predicts that a verbal report should impose a stronger reduction than private attention alone because speech requires lexical selection, syntactic encoding, working-memory stabilization, and social commitment. EEG, MEG, fMRI, or intracranial recordings should show a stronger decrease in representational entropy or manifold dimensionality after spoken report than after silent introspection. The focus-of-attention and awareness-buffer accounts provide convergent cognitive reasons to expect capacity-limited stabilization, while neural state reduction predicts that the magnitude and content of the reduction should depend on the report basis (Cowan et al., 2025; Allen et al., 2026).

### 7.3 Prediction 3: ambiguous conscious states should show interference-like probability structure

When candidate thoughts overlap semantically or affectively, report probabilities should deviate from classical additivity. This can be tested by comparing reports of individual thoughts, combined prompts, and order-manipulated prompts. If interference terms are present, the probability of reporting combined or related thoughts should depend on contextual phase-like relations rather than on simple additive probability.

### 7.4 Prediction 4: neural decoding should improve when measurement context is modeled explicitly

Decoders of ongoing thought should improve when they include not only latent neural activity but also the question or report context. A report of “worry” after an affective prompt and a report of “planning” after a task prompt may arise from overlapping latent states but different measurement bases. Modeling the basis should improve the prediction of report outcomes.

### 7.5 Prediction 5: report should stabilize a lower-dimensional neural manifold

Before reporting, neural activity associated with ongoing thought should occupy a broader and more metastable state space. During and after the report, the activity should contract toward a lower-dimensional, more stable manifold associated with the selected content, language encoding, and response preparation. This can be tested using representational similarity analysis, manifold learning, neural state-space modeling, and temporal generalization.

### 7.6 Prediction 6: repeated self-observation should alter thought dynamics

Repeated introspective probing should change the subsequent trajectory of thought. In quantum-inspired models of bistable perception, repeated observation provides a formal precedent for Zeno-like stabilization (Atmanspacher et al., 2004), although the present prediction concerns ordinary cognitive probing rather than physical quantum dynamics. Repeated self-observation may stabilize certain categories of self-description and suppress others. Experience-sampling studies can test whether frequent thought probes change the distribution, granularity, or emotional valence of subsequent reports.

### 7.7 Prediction 7: leading linguistic frames should alter immediate and delayed report states

If a probe participates in state reduction, semantically leading wording should shift not only immediate response probabilities but also, under some conditions, the later memory state from which subsequent reports are generated. This can be tested by presenting controlled events or autobiographical prompts, varying neutral versus leading question wording, and measuring immediate report, delayed recall, confidence, and neural representational change. The prediction is motivated by reconstructive-memory findings such as Loftus and Palmer (1974), but the effect is not uniquely quantum-like. Neural state reduction would receive distinctive support only if basis-specific order effects or non-additive probability structure improves predictive accuracy beyond response bias, demand characteristics, and classical reconstructive-memory models.

## 8 Empirical research program

A testable theory should specify how it could be falsified. Neural state reduction can be tested through four empirical programs.

### 8.1 Program 1: randomized introspective question-order and wording studies

Participants can be interrupted during rest or task performance and asked introspective questions in randomized order. For example:

1. What are you thinking?
2. What are you feeling?
3. What are you trying to do?
4. What image, word, or memory is most present?

The model predicts systematic order effects beyond ordinary response noise. Classical models that treat each question as sampling a pre-existing independent variable should underpredict these effects. A complementary wording manipulation should compare neutral and leading probes, followed by a delayed retest, to determine whether the measurement context changes only the response criterion or also the later report-relevant state. Demand characteristics, social desirability, and source-monitoring error must be measured explicitly.

### 8.2 Program 2: neural entropy before and after the report

The EEG, MEG, fMRI, or intracranial recordings can measure neural state-space dimensionality before and after the report. The model predicts a reduction in report-relevant entropy and a suppression of representational ambiguity during report stabilization. The strongest version of the effect should occur for spoken report, followed by typed report, button press, silent labeling, and unprobed mentation.

### 8.3 Program 3: no-report versus report-based consciousness paradigms

No-report paradigms can distinguish latent conscious dynamics from report-stabilized dynamics. Neural state reduction predicts that no-report conditions should preserve richer latent dynamics, whereas report conditions should add a state-reduction signature associated with attention, working memory, language, and response selection. This prediction complements the no-report literature by providing a formal account of what the report adds.

### 8.4 Program 4: semantic interference and conceptual ambiguity

Participants can be asked to report ambiguous thoughts, emotional mixtures, or metaphorical associations. Quantum-like models should be compared against classical mixture models. Evidence for non-additive probability structure, response replicability constraints, and basis-dependent reports would support the model. Failure to find order effects, interference, or entropy reduction under controlled conditions would weaken it.

## 9 Relationship to existing theories

Contemporary theories of consciousness address different explananda and levels of analysis, including phenomenal character, access, integration, recurrence, metacognitive representation, predictive control, and physical realization (Seth and Bayne, 2022). Neural state reduction is not proposed as a complete theory of phenomenal consciousness. It is a formal account of how a latent, report-relevant neural-cognitive state becomes selected, stabilized, and updated under a question or report context. The comparisons below, therefore, distinguish compatibility at the level of access and report from stronger claims about the physical basis or metaphysics of consciousness.

### 9.1 Embedded processes and awareness-buffer models

Cowan et al. (2025) proposed that consciousness is organized around a capacity-limited focus of attention embedded within activated long-term memory, with the focus shaped by both executive control and the bottom-up draw of salient or thematically relevant representations. Allen et al. (2026) placed an awareness buffer at the heart of working memory as a multidimensional interface for conscious storage, binding, manipulation, and control. These accounts specify cognitive architecture: where candidate information is activated, how much can be focused, and how attention is controlled.

Neural state reduction is complementary. It does not redescribe activated memory or the focus of attention with quantum terminology. It formalizes how a particular question, decision, or report demand defines a basis, changes the selection problem, and updates the state after an answer. The frameworks make overlapping but separable predictions. Embedded-process and awareness-buffer models predict capacity limits and control effects; neural state reduction additionally predicts non-commutative order effects, contextual interference, and basis-dependent state updates. Their combination suggests direct model comparisons in which activated-memory strength, focus-of-attention occupancy, executive control, and report basis are manipulated independently.

### 9.2 Global neuronal workspace

Global neuronal workspace theory argues that conscious access occurs when information is amplified and made globally available to multiple systems (Baars, 1988; Dehaene and Changeux, 2011; Mashour et al., 2020). Neural state reduction is compatible with this view but focuses on a different part of the problem. GNW explains how information becomes globally accessible; neural state reduction describes how a latent neural-cognitive state becomes selected under a report context. In this sense, state reduction may be one formal mechanism by which access and report stabilize content.

### 9.3 Integrated information theory

Integrated information theory (IIT) begins from phenomenological axioms and asks what physical systems could support integrated experience (Tononi et al., 2016). Neural state reduction does not attempt to replace IIT. It is more modest and more operational: It models the transition from latent conscious potential to reportable content. It is therefore most directly relevant to reportability, metacognition, and the structure of introspective measurement.

### 9.4 Higher-order, recurrent-processing, and attention-schema theories

Higher-order theories relate conscious awareness to higher-order representations of mental states, recurrent-processing theories emphasize recurrent neural interactions rather than feedforward processing alone, and attention-schema theory proposes that the brain constructs an internal model of attention that supports awareness judgments and reports (Graziano and Webb, 2015; Lamme, 2006; Lau and Rosenthal, 2011). Neural state reduction does not adjudicate which of these processes is necessary or sufficient for phenomenal consciousness. It models the basis-dependent selection and state update that occur when available contents must be rendered as an explicit answer. These theories can therefore supply candidate mechanisms or representations within the latent state without being identical to the projection formalism.

### 9.5 Orchestrated objective reduction (Orch OR)

Orchestrated objective reduction (Orch OR) proposes that conscious moments arise from orchestrated quantum processes in neuronal microtubules and objective reduction of quantum states (Hameroff and Penrose, 2014). Neural state reduction is conceptually distinct. It does not require microtubular quantum computation, sustained biological quantum coherence, or gravitationally induced objective reduction. Its use of “state reduction” is computational: A report context selects and updates a latent cognitive state. Orch OR is, therefore, a quantum-biophysical theory of phenomenal consciousness, whereas neural state reduction is a quantum-inspired formal theory of context-dependent reportability.

### 9.6 Dynamic organicity theory and recent work by Poznanski

Poznanski’s Dynamic Organicity Theory and its recent extensions—Embedded Quantum Physicalism and Processual Relational Geometry—develop a multiscale, non-reductive physical account in which intrinsic information, quantum-thermal processes, self-referential functional geometry, and trans-scale closure are proposed as constitutive of phenomenality (Poznanski, 2024, 2025, 2026). Neural state reduction does not adopt these substrate-level or ontophysical commitments. It is agnostic about whether the latent-to-report transition is implemented by entirely classical neural dynamics or by mechanisms that include quantum-biophysical effects. The empirical interface is explicit: Variables derived from these multiscale frameworks would need to improve prediction of basis dependence, order effects, latent-state dynamics, or report-stabilization signatures.

### 9.7 The hard problem and the meta-problem of consciousness

Chalmers (1995) distinguished functional problems of discrimination, access, control, and report from the hard problem of why physical processing is accompanied by subjective experience. Chalmers (2018) later framed the meta-problem as explaining why systems make judgments and reports about consciousness. Neural state reduction is directed at the functional and report-related domain: It models how report-relevant multiplicity becomes a stabilized answer and how the act of reporting changes the subsequent state. It does not claim that explaining this transition explains why experience exists. Because it formalizes the construction and stabilization of introspective reports, it may contribute to the meta-problem without claiming to dissolve the hard problem.

### 9.8 Predictive processing and active inference

Predictive-processing and active-inference frameworks model perception, cognition, and action as inference under uncertainty within generative models (Clark, 2013; Friston, 2010). Neural state reduction is compatible with this tradition but emphasizes the special role of measurement context. A question does not merely reveal the posterior distribution; it can change the hypothesis space and the basis in which the posterior is expressed. The two frameworks can be integrated by treating the report as an active-inference policy that reduces uncertainty in a socially and linguistically constrained state space.

### 9.9 Attractor and metastable-state models

Attractor-network and metastable-state models describe how neural systems settle into stable patterns or transiently move among quasi-stable states (Hopfield, 1982; Rabinovich et al., 2008). Neural state reduction can be interpreted as a formal description of context-driven attractor selection. The pre-report state occupies a metastable landscape; the report context reshapes the landscape; the system settles into a reportable basin. The quantum-inspired formalism adds non-commutativity, interference, and basis dependence to the attractor metaphor.

### 9.10 Embodied and cross-cultural perspectives

Broader traditions emphasize that conscious experience is not exhausted by propositional thought or cortical report. Lu et al. (2022), drawing on traditional Chinese medicine, argued for a wider view in which conscious experience is deeply connected to bodily and organ-related processes. Neural state reduction does not adopt specific traditional Chinese medicine mechanisms, nor does it treat organ systems as quantum components. It accepts the broader methodological point that interoceptive, affective, visceral, and culturally structured contexts can shape the candidate state and the basis of the report. In the present formalism, these influences enter through ${\hat{H}}_{\text{affect}}$ , embodied components of the latent state, and contextual stabilization operators. This extension converts embodiment from a metaphysical assertion into a source of testable context dependence.

## 10 Discussion

The central contribution of this manuscript is a formal reframing of conscious report. Rather than treating an introspective report as a passive window into an already determinate thought, neural state reduction treats the report as a measurement-like transformation. The latent neural-cognitive state contains multiple candidate contents. A report context selects a basis. The selected outcome updates the state. Subsequent reports are therefore conditioned by prior reports.

This framework helps explain a common phenomenological fact: People often discover what they think by saying it. This is not necessarily self-deception or poor introspective access. It may be a structural feature of cognition. Speech and report convert diffuse latent states into discrete communicable states. The act of expression is: not downstream of thought alone; it is part of thought stabilization.

The working-memory literature clarifies what this claim does and does not add. Cowan et al. (2025) provided an architecture in which a small focus of attention is embedded within a wider activated-memory field and controlled by both executive goals and representational draw. Allen et al. (2026) identified a consciously accessible awareness buffer that integrates multidimensional content and control. Neural state reduction adds a formal account of how the eliciting context changes the selection basis and the post-report state. Cowan and Rachev’s (2018) historical analysis further showed that a broad field narrowing into a focus has roots in Wundt’s experimental approach to consciousness. The present contribution is, therefore, not the field-to-focus idea itself but a context-sensitive mathematical account of reduction, order, and update.

The framework also helps clarify why consciousness science struggles with reports. If a report changes the state being measured, then report-based experiments cannot be interpreted as pure readouts of pre-report consciousness. Conversely, no-report experiments may miss the specifically human capacities of metacognitive articulation, linguistic stabilization, and self-description. Both are necessary. The key is to model a report explicitly rather than treating it as a transparent output channel.

The reconstructive-memory literature adds a crucial safeguard. Questions can stabilize reports that are distorted or false, as demonstrated by wording effects on later memory reports (Loftus and Palmer, 1974). Neural state reduction, therefore, does not equate stabilization with truth. It predicts a context-sensitive outcome, not privileged access to an objective mental record. Experiments must distinguish genuine state change from response bias, compliance, demand characteristics, source-monitoring error, and ordinary Bayesian reconstruction.

The quantum-inspired formalism is useful because it provides mathematical tools for exactly these problems. Classical probability can represent uncertainty over thoughts, but it does not naturally represent the fact that questions can be incompatible, that order can matter, that candidate meanings can interfere, and that measurement can change the state. Quantum probability was designed for systems in which measurement context matters. The updated synthesis of Busemeyer and Bruza (2024), including its treatment of contextuality, dynamics, and open systems, supplies the most direct formal foundation for this use.

The theory also has practical implications for neuroscience and artificial intelligence. In neuroscience, it suggests new analyses of neural manifold contraction, entropy reduction, and context-dependent decoding during introspection. In AI and cognitive modeling, it suggests that language outputs may be treated as measurement-like reductions of high-dimensional latent embeddings. Large language models are not conscious, but they provide a useful engineering analogy: A high-dimensional latent distribution becomes one token sequence only after sampling, decoding constraints, and context are applied. Human thought is biological and embodied, but the computational pattern of latent potential becoming a reportable sequence may be shared across systems.

## 11 Limitations

The framework has several limitations. First, it is a hypothesis-and-theory manuscript, not a report of new biological recordings. The formalism establishes conceptual coherence and generates tests, but it does not demonstrate that a neural circuit implements the proposed decomposition.

Second, the simulations are synthetic and illustrative. They demonstrate formal consequences but do not validate the model biologically. The entropy and coherence reductions are partly built into the selective projection operation, and real data should show partial, noisy, and task-dependent stabilization rather than ideal collapse.

Third, the mapping between Hilbert-space constructs and neural mechanisms remains provisional. The latent state may include neural activity, activity-silent memory, synaptic states, embodied signals, or abstract representational variables. The theory must specify which measurement level is appropriate for each experiment.

Fourth, the model does not solve the hard problem of consciousness or explain why neural activity is accompanied by subjective experience. It addresses the computational transition from latent report-relevant structure to an explicit report.

Fifth, the report is not the only form of state stabilization. Action, perception, affect, interoception, and bodily regulation may also reduce or reshape latent cognitive states. Broader embodied perspectives therefore define an extension of the model, not a completed mechanism.

Sixth, the model must be compared against strong classical alternatives, including Bayesian, predictive-processing, dynamical-system, attractor-network, working-memory, reconstructive-memory, and signal-detection models. Leading-question and false-memory effects are not uniquely quantum-like. The framework earns support only where non-commutative, interference-based, or basis-dependent models provide reliable out-of-sample gains under matched complexity.

Seventh, state reduction does not imply veridicality. A stabilized report can be accurate, approximate, socially shaped, or false. Experiments must separate contextual state change from response criterion shifts, compliance, source-monitoring failure, and linguistic suggestion.

These limitations define the next stage of work. The model should be judged by whether it generates predictions that are clearer, more quantitative, or more empirically successful than existing alternatives.

## 12 Conclusion

Neural state reduction proposes that conscious thought becomes reportable through a measurement-like transformation. Before reporting, the neural-cognitive system may occupy a high-dimensional latent state containing multiple partially active candidate thoughts and activated memory representations. Attention, questioning, decision, and speech impose a context-specific basis that reduces this latent state into a reportable cognitive outcome. The theory is quantum-inspired but not quantum-biophysical: It uses the mathematics of state vectors, density matrices, projection operators, non-commutativity, interference, and contextual state updates to model cognition, not to claim that the brain is a quantum computer.

The resulting framework offers a testable bridge between quantum cognition and computational neuroscience while remaining compatible with embedded-process and awareness-buffer theories of working memory. It predicts introspective order effects, report-induced entropy reduction, interference among ambiguous thoughts, improved neural decoding when measurement context is modeled, contraction of neural state spaces during verbal report, and wording-dependent reconstruction that must be distinguished from uniquely quantum-like effects. If supported empirically, the framework would clarify why introspection is both informative and constructive: when we are asked what we are thinking, we do not simply observe a thought already sitting fully formed in the mind. We participate in making one thought reportable.

## Data Availability

The original contributions presented in the study are included in the article/supplementary material, further inquiries can be directed to the corresponding author.
